# A Fractal-Based Authentication Technique Using Sierpinski Triangles in Smart Devices

**DOI:** 10.3390/s19030678

**Published:** 2019-02-07

**Authors:** Adnan Ali, Hamaad Rafique, Talha Arshad, Mohammed A. Alqarni, Sajjad Hussain Chauhdary, Ali Kashif Bashir

**Affiliations:** 1 Faculty of Computing and Information Technology, University of Sialkot, Sialkot 51040, Pakistan; hamaadrafique@gmail.com (H.R.); talhaa.aarshad@gmail.com (T.A.); 2 College of Computer Science and Engineering, University of Jeddah, Jeddah 21577, Saudi Arabia; maaalqarni8@uj.edu.sa (M.A.A.); shussain1@uj.edu.sa (S.H.C.); 3 School of Computing, Mathematics, and Digital Technology, Manchester Metropolitan University, Manchester M15 5RN, UK; dr.alikashif.b@ieee.org; 4 Faculty of Science and Technology, University of the Faroe Islands, FO-100 Tórshavn, Denmark

**Keywords:** Authentication, shoulder surfing, graphical password scheme, fractal, Sierpinski triangle, smart devices, J0101

## Abstract

The prevalence of smart devices in our day-to-day activities increases the potential threat to our secret information. To counter these threats like unauthorized access and misuse of phones, only authorized users should be able to access the device. Authentication mechanism provide a secure way to safeguard the physical resources as well the information that is processed. Text-based passwords are the most common technique used for the authentication of devices, however, they are vulnerable to a certain type of attacks such as brute force, smudge and shoulder surfing attacks. Graphical Passwords (GPs) were introduced as an alternative for the conventional text-based authentication to overcome the potential threats. GPs use pictures and have been implemented in smart devices and workstations. Psychological studies reveal that humans can recognize images much easier and quicker than numeric and alphanumeric passwords, which become the basis for creating GPs. In this paper a novel Fractal-Based Authentication Technique (FBAT) has been proposed by implementing a Sierpinski triangle. In the FBAT scheme, the probability of password guessing is low making system resilient against abovementioned threats. Increasing fractal level makes the system stronger and provides security against attacks like shoulder surfing.

## 1. Introduction

Smart devices have become an intrinsic part of modern life. These devices store our sensitive information like Personal Identification Numbers (PINs), contact details, pictures, important documents, and financial information [1]. Such information needs to be protected to provide security and privacy to the user. A consumer report in the USA revealed that 34% of smart device users either use simple code to lock their devices or have no security mechanism at all. The report further says that 36% users use a basic lock for the security of their phone (4-digit PIN lock) [2]. It has been observed from real-time events that many text-based passwords are not enough and can be easily breached to access sensitive data. For example, British Broadcasting Corporation (BBC) reported that Superdrug online store [3], last.fm [4] and Ticketmaster [5] in the United Kingdom (UK) had all suffered from password breaches, therefore, sensitive data remain vulnerable [2]. User authentication is considered a first step to prevent a data breach.

Different authentication schemes are proposed in the literature for data protection. Among them, the most commonly used traditional password technique is text-based password authentication [2]. This scheme is preferred because password combinations are easy to remember and can be converted into any unique password combination through exchange protocols [6]. It can consist of public parameters like name, family name, city name, children name, a PIN, date of birth (DoB), the first six letters of common English type-writer/keyboard (QWERTY) or commonly used words, etc. However, this scheme is vulnerable to dictionary attacks, brute force attacks, and guessing attacks [7]. To protect their devices against these attacks, end-users are recommended to use lengthy and complex passwords with combinations of letters and numerals, e.g., upper-case as well as lower-case letters, numbers, and special characters. Complex passwords are usually lengthy and difficult to remember; therefore, users tend to write them down on paper, save them in Google accounts for future logins or in an electronic file, which increases the risk of theft that will lead to security breaches [2].

To overcome the issue of complex passwords, various graphical password (GPs) schemes were proposed in the literature [8,9]. The GPs like Passface [10], Passhint [11], etc. makes the smartphones more compatible rather than the traditional ones as they provide a large symbol space over text-based password techniques. Therefore, they are being adopted in smart devices [2]. Research revealed that users pay more attention to technology which is easy to use [12]. The GPs schemes consist of images and icons. Users must select images or icons from the pool of images to create a password. These selected images and icons will be used for password authentication. Despite these benefits, all the present graphical schemes are still vulnerable to different attacks, e.g., brute force attacks, shoulder surfing, and smudge attacks [1].

**Figure 1:**
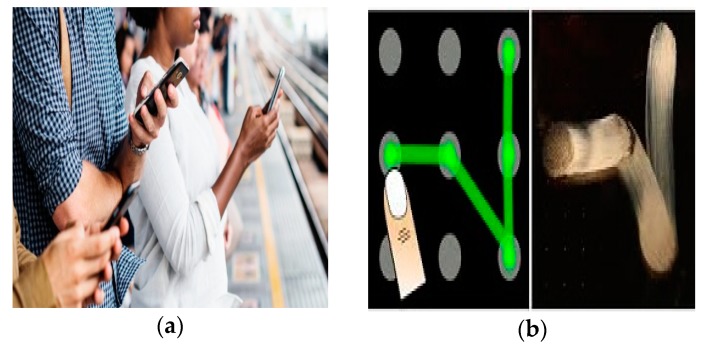
(**a**) Shoulder surfing attack; (**b**) smudge attack.

Although GPs provides advantages over text-based password as a user does not need to remember long passwords, it is based on images and patterns, etc., besides that, most of the GPs use a limited password space that helps attackers in using brute force to break them [13]. Though GPs help in improving security, they are however still vulnerable to shoulder surfing, which is defined as” password observation by glancing over the shoulder or by eavesdropping on private conversations” [2] as shown in Figure 1a. It is the most common method of stealing a password in a crowded place, as it is easy to stand right next to a smartphone user [14]. Another mechanism for accessing passwords is by recording through high definition cameras while a user is entering a password on a smart device [2]. To overcome the drawbacks of GPs, user scan use pattern-based authentication schemes [1]. These were introduced to overcome shoulder surfing. However, a pattern-based authentication scheme is also vulnerable to smudge attacks, another prominent type of attack described in the literature [13], which is defined as “oily residue left on the screen used to crack passwords“ [2], shown in Figure 1b. Whenever the user enters the password, smudges or oily residues remain on the display helping the attacker easily determine the passwords by viewing the smudges on the screen at a different angle, e.g., 60° [13]. Therefore, eliminating smudge attacks is considered difficult. With different light angles or camera angles, an attacker can identify the smudges on the screen, which will help them unlock the password patterns. A study shows that there is a68% chance that an attacker will fully unlock a device and a 98% chances of partial unlocking [13]. Most of the currently proposed GPs are vulnerable and do not tackle the attacks explained in Section 2. Therefore, finding a solution to these attacks is still an active research area. For the security of sensitive information and data, it is recommended that users follow several password creation rules recommended by various researchers for authentication purposes [15,16,17]. Some of the popular password creation rules are as follows:

(1) A password should have at least eight characters as this will increase the password space explained in Ref. [15] i.e., password keys with the difficulty in breaching it.
(2) Words found in a dictionary should not be used in password creation because of their common nature. They are widely employed by users and can be easily breached by making the password weak.
(3) A majority of users either write down their password or save them in the cloud which will increase the risk of cracking. Therefore, a password should not be saved or written down anywhere, which will create complexity and difficulty for the attackers to breach it.
(4) Changing passwords periodically will increase password strength. For example, if someone is observing users’ fora long time particularly close friends of users, they might get familiar with the same password. Therefore, changing the password periodically will increase the password security.
(5) A password generated randomly will be considered as a strong password. It is because the user usually selects common keywords or patterns for security purposes. Random password generation helps the user in creating complex passwords which might be a combination of alphanumeric words or random images with strong security parameters.
(6) Every system or account should be protected with a different password, so if an attacker is successful in cracking one password, he will not be able to breach other accounts.

For the purpose of ease of remembrance, users often ignore these rules, which results in password risk. Therefore, the password should not contain publicly known parameters and must follow the password generation rules.

**Figure 2:**
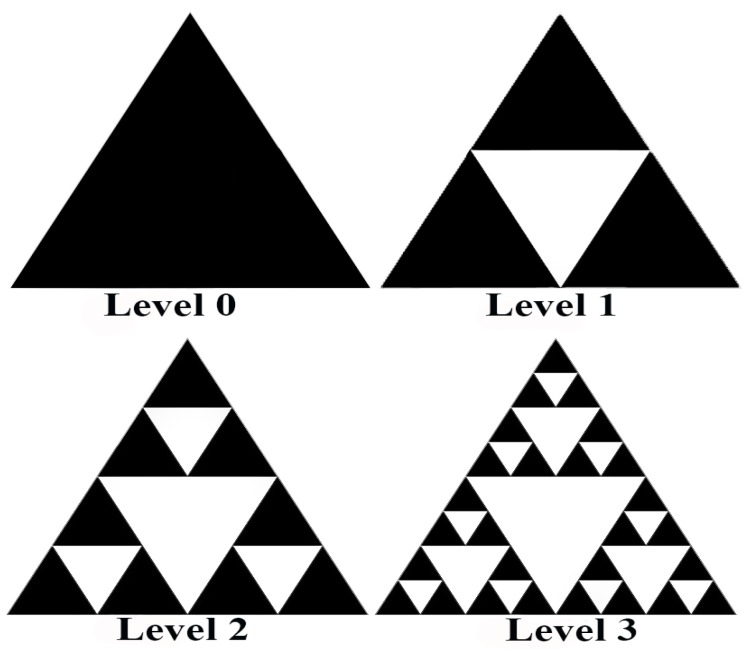
Levels of Sierpinski triangles

In this research paper, we propose a novel FBAT based on Sierpinski triangles. We have developed this technique with a combination of graphical and pattern-based schemes. It consists of various levels and at each level, the user must select a triangle with a color combination to create a password as shown in Figure 2. The proposed technique is helpful in avoiding password breaches, as it is stronger than traditional text-based passwords or PIN numbers; similarly, this technique creates complexity for breaching the password because of the reduced probability of successfully breaching the password, as explained in Section 4. Simulation results proved that the proposed scheme is resilient to (i) brute force attack, (ii) shoulder surfing and (iii) smudge attack and much stronger than traditional text-based password and 4-digit PIN password. FBAT also proved the ease of use during password creation and login phase. 

This paper is divided into the following sections: A detailed introduction of our research idea and problems is given in Section 1. Related work supporting our problem is described in Section 2. The proposed FBAT scheme based on Sierpinski triangles to mitigate the problems of shoulder surfing, brute force and smudge attacks is briefly discussed in Section 3.A systematic analysis is conducted in order to evaluate the usability and memorability of the system from the user perspective in Section 4. Practical implementation and evaluation of the proposed technique with the help of a user study is presented in Section 4. Results obtained from the user study are briefly discussed is Section 5. A security analysis with respect to the existing techniques is performed in Section 7. Finally, paper is concluded with the outcomes of the proposed technique in Section 8.

## 2. Related Work

Passwords are used to secure the data saved on smart devices [2]. Users either employ traditional password techniques like text-based password or graphical password (GPs) schemes for securing their private data [15]. Based on this an extensive number of GPs has been proposed in the literature. The concept for GPs was introduced because of the quicker recognition of graphical images rather than traditional passwords by human beings [2]. GPs are divided into four categories: (i) pure recall-based schemes, (ii) recognition-based schemes, (iii) cued recall-based schemes, and iv) hybrid schemes [18]. Pure recall base schemes are defined as “repetition of registered drawings known as drawn metric system” [19]. Few examples of pure recall base schemes are Draw-A-Secret (DAS) [19], pass-go [20] and Android Pattern Lock (APL) [21]. Hybrid schemes like VibraPass [22] and VAP Code [2] are “a combination of multiple authentication schemes” [2]. However, our proposed novel graphical pattern-based scheme is a combination of recognition and cued recall-based schemes. Therefore, in this paper, we focus only on recognition-based and cued recall-based schemes.

### 2.1. Recognition-based Scheme

In a recognition-based scheme, a user must recognize preselected series of images which are used to create a password. Recognition-based schemes are easy to remember and easy to identify by the user. On the other hand, the system efficiency is reduced by the need to process a larger set of images or icons, e.g., 1000 or more [15]. 

The graphical techniques work by selecting different images or icons from a database for creating a password. The user must remember the selected images during the authentication phase. Some of the recognition-based schemes are elaborated in this section. In Ref. [10], the authors proposed the PassFaces technique that uses a human face verification technique for login. In this scheme, the user has to select a number of images in the password creation process. While in the login phase the user must tap the selected image from among several decoy images. The drawback of this scheme is the natural inclination to select faces based on cultural, race or gender preferences. The idea of [10] was extended by [23] through changing the concept of images to stories. In this scheme, the user must select routine images instead of a human face in a story formation such that selected images will appear in the sequence in which it was captured. This sequence of images will make a story of that event from which images were taken. However, the drawback of this technique is that users may select family events or university or school life events which may be familiar to their friends. In Ref. [11], researchers proposed the Passhint authentication system, in this scheme, the user has to select four images and then create a hint for them in the registration phase. In the authentication phase, hints will be used to recognize the targeted images among 15 decoy images. The limitation of this scheme is that a user must memorize pictures as well as hints for recognition of the password which increases the login time. In Ref. [7], the author proposed a text-based graphical password scheme where the user selects five images from among sixteen random images. Displayed random images are a combination of graphical and text-based images which are used to create a password. The login phase of this scheme consists of a 4 × 3 grid, in which the last vertical grid is continuously moving. When the selected image in the moving grid is aligned with the other two selected images in the static grid, then the user will tap on the image to unlock the device. The wait time for the alignment of images in this scheme results in an increased login time. 

### 2.2. Cued recall-based Scheme

In cued recall-based graphical scheme, users must click on some pre-selected points on given images to unlock the password. The graphical password technique was first proposed by Blonder in 1996 [24]. In his technique, users have to click on an already defined location on an image. It is done for the authentication of the users. The Blonder technique as extended in Ref. [14]is known as a PassPoint system. This system gives free hand to users to click anywhere on the picture to create their password. Research conducted by [25] proposed a cued click point technique. In this technique, the user clicks on one point per image in the sequence of images. Password generated in this technique was generated on clicking different images which were shown in a sequence. This study reports higher security than PassPoint. As discussed, the aforementioned literature schemes are vulnerable to smudge, shoulder surfing and brute force attacks, either fully or partially. 

Generally, users use a simple text-based password with common keywords or graphical passwords with personal images which can be easily tracked or observed by the attacker creating security breach issues. Therefore, our proposed FBAT will overcome the issues of both text-based password and graphical passwords. FBAT will use the concept of the recognition-based scheme and cued recall-based scheme. The user will recognize their selected colored triangle and then will use the concept of the cued recall-based scheme for clicking on the pre-selected triangle on each level. Hence, our proposed scheme will provide better resilience against shoulder surfing, smudge and brute force attacks as compared to other techniques.

## 3. Proposed Methodology

The methodology section explains the proposed technique, i.e., FBAT, it is developed in order to mitigate the attacks that might breach the security of smart devices, e.g. shoulder surfing, brute force and smudge attacks, by combining the benefits of recognition-based and cued recall-based authentication schemes.

Our proposed graphical authentication scheme is known as FBAT. In the FBAT scheme a pattern is generated using fractals, i.e., Sierpinski triangles, also called Sierpinski gaskets or Sierpinski sieves, as shown in Figure 2. The FBAT scheme is based on a repetition of triangles. In the Sierpinski triangle, a selected pattern with the help of a color scheme is saved in the device storage which is then retrieved from the device storage during the authentication phase. Level 1 of FBAT comprises four equilateral triangles inscribed in it. Three of them are solid triangles and one is empty. We can also describe it as removing the center triangle or putting an empty triangle in the center which will create three more equilateral triangles.

As shown in Figure 2, there is Level 0 which is only one triangle. When we remove the triangle from the center, it will give three more triangles like in Level 1. This makes a total of four triangles, three filled black color triangles and one empty one. If we keep on removing triangles from the filled triangle, then we move to Level 2. In Level 2 there are nine triangles in black and four empty triangles. However, our concern is with filled triangles. If we keep putting an empty triangle in every black triangle, then the levels will keep on increasing. Level 3 has 27 filled triangles. The number of triangles increases by 3^n^ (n = 0, 1, 2, 3, 4, …, n) where n is the level. At the 4th level, there would be 3^4^ = 81 triangles, at the 5th level 3^5^ = 243 triangles, and this value will keep on increasing with rising level. This is known as the password space. It is defined as “number of password arrangements presented for use by users in a system” [14]. We can calculate password space through ϐ^¢^ as shown in Table 1. FBAT will use the Sierpinski triangle as a password hiding technique. It can be used in mobile applications and operating systems as a pattern lock and passwords technique [2].

### 3.1. Registration of Pattern

**Figure 3:**
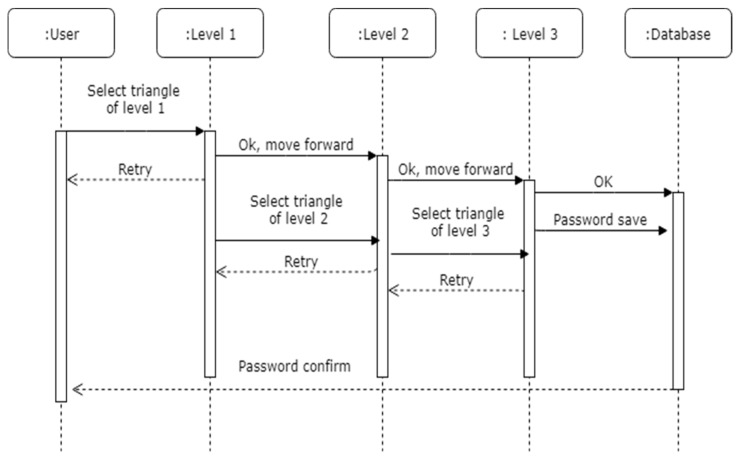
Sequence diagram for password registration and verification.

The registration process is explained in Figure 3 with the help of a sequence diagram and the workings of the technique for registration can be found in Algorithm 1. The notations used in the pseudo code are explained in Table 2. In the registration phase, the user must select the pattern which they want to use as a password in our developed prototype with the help of colored triangles. These selected color triangles of each level will be considered as a password, which user has to memorize for the authentication phase.

**Figure 4:**
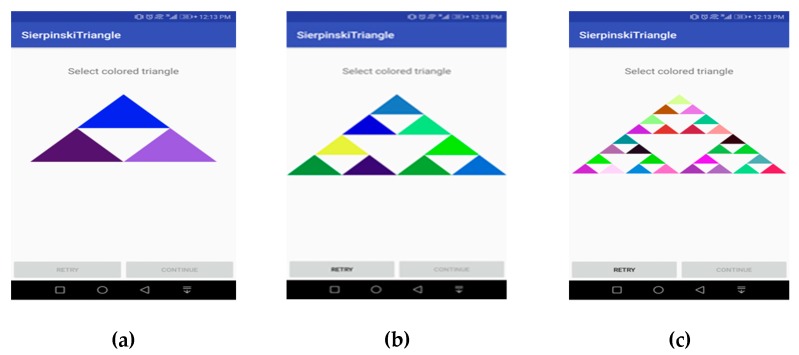
(**a**) Registration phase level 1; (**b**) registration phase level 2; (**c**) registration phase level 3.

We have fixed the number of Sierpinski levels at three because of limited screen size, as seen in Figure 4, because with higher levels, the area of triangles will keep reducing and that may lead to fat finger problems, as it is a property of the Sierpinski triangle that when the level increases, the area of each triangle reduces, and perimeter increases. Therefore, we don’t recommend increasing the levels of Sierpinski triangles in handheld devices as they have limited screen size. Hence FBAT is fixed to Level 3 in handheld devices. However, Sierpinski triangles above Level 3 could be implemented in a device where the screen size is larger than 10 inches.


**Algorithm 1. Registration Phase of FBAT**
1: Draw a GUI in function 2: Initialize Ю 3:     Select triangle ← {L1} 4:     Pattern saved ← ϸ 5:     L1++ 6:  If 7:  Wrong selection ← {L1} 8:     Retry 9:     Return L1 10:  Else 11:     Select triangle ← {L2} 12:     Pattern saved ← ϸ 13:     L2++ 14:  Endif 15:     Wrong selection ← {L2} 16:      Retry 17:      Return {L2} 18:  Else 19:      Triangle selection ← {L3} 20:      Data saved ← ϸ 21:  Endif 22:      Wrong selection ← {L3} 23:      Retry 24:      Return {L3} 25:  Else 26:      Continue for confirmation 27:  Endif 28:      Repeat L1 to L3 29:       Confirm 30:  If 31:      Set password == confirm password 32:      Password set 33:       Password saved ← ∂ 34:  endif

**Figure 5:**
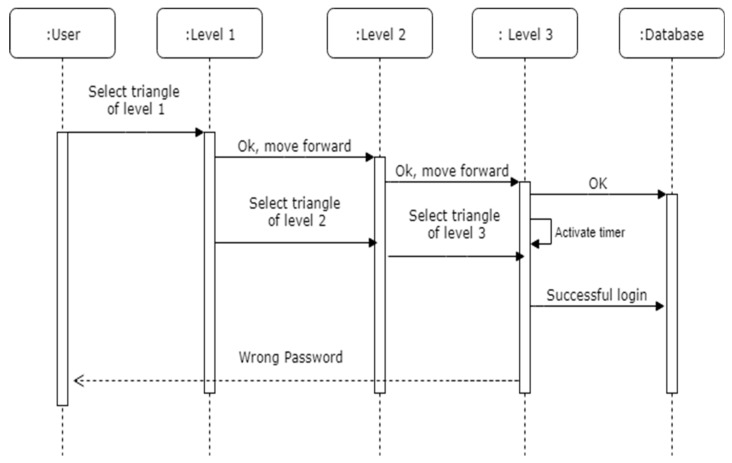
Sequence diagram for password authentication.

**Figure 6:**
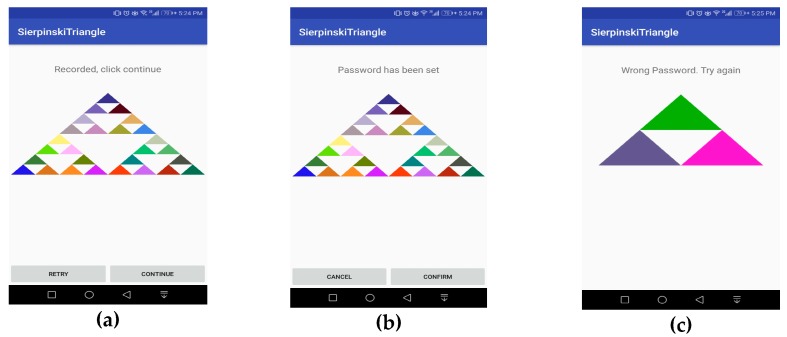
(**a**) Password recorded; (**b**) password set; (**c**) wrong password.

Password creation using FBAT is explained with the help of few steps which are explained here:

(1) Level 1: In this step, the level appears in front of the user as shown in Figure 4a. It consists of three colored and one empty triangle. Here the user selects one colored triangle from n (i.e., 3) numbers of triangles. At this stage, the selection probability of a password is 0.3333 as seen in Equation (1).
(2) Level 2: In the second step, a new level appears and Level 1 disappears, as shown in Figure 4b. It consists of nine colored triangles. At this stage, the user selects one triangle from n (i.e., 9) number of triangles, with a probability of 0.037037 as shown in Equation (2).
(3) Level 3: In the third step, a level appears, and Level 2 disappears as shown in Figure 4c. This level consists of 27 colored triangles. As done in the previous steps, the user selects one colored triangle from among n (i.e., 27) triangles with a probability of 0.001371 as shown in Equation (3). Sequence of performing authentication is elaborated in sequence diagram in Figure 5. After this level, the user presses the confirm button which saves the pattern as shown in Figure 6a.
(4) The user repeats these steps to confirm the password as seen in Figure 6b. After confirmation of the pattern, it was set.

### 3.2. Authentication

The pattern which was created and stored in the registration phase is used in the authentication phase. In this phase, the user has to select the pattern in order to authenticate himself. However, all the colored triangles in every phase will randomly shuffle themselves, so that the colored pattern that was created during the password creation phase will not appear on same place at which it was during registration phase. The user will select the pattern which was created during password creation from levels 1, 2 and 3. Each time the user selects a triangle from the given pattern, the current level will disappear, and a new pattern level will appear. Soon after completing the correct pattern, the device will unlock itself. On failure, the user will be redirected to Level 1 as shown in Figure 6c. Five continuous failed attempts will lock the device. The flowchart for the authentication phase is defined in Figure 5. The working of the authentication is explained in the algorithm below.


**Algorithm 2. Authentication Phase of FBAT**
1: Draw a GUI in function 2:   Initialize Ю 3:   Initialize ς 4:      Select triangle {L1} 5:      Pattern saved ϸ 6:      L1++ 7:       Select triangle {L2} 8:       Pattern saved ϸ 9:       L2++ 10:        Triangle selection {L3} 11:        Data saved ϸ 12:   if 13:       Pattern value = = saved value 14:       Unlock 15:   Else 16:       Error message 17:       Retry 18:       ς ++ 19:   if 20:       ς ++ ≥ 5 21:       Timer set == 30 s 22:   endif

## 4. System Analysis

Our novel technique provides strong resilience against brute force, shoulder suffering and guessing attacks. Because of its large password space and complexity levels, it is very difficult for machines or human beings to break this combination.

The multiplication rule of probability says that “the probability that events A and B both occur is equal to the probability that event A occurs times the probability that event B occurs, given that A has occurred”. Because it works in a manner that, first we select triangle from Level 1 then 2 and 3 and so on. In this case first event that is a selection of a triangle in level one occurs, and after that second event that is a selection of a triangle from level two will occur. This will continue until the password is created. According to the rule of multiplication of probability P(A∩B) = P(A)P(A|B) we have the following equations:

P(Level1) = 1/3 = 0.3333(1)

P(Level2) = 1/3 × 1/9 = 0.037037(2)

P(Level3) = 1/3 × 1/9 × 1/27 = 0.001371(3)

P(Success) = 0.13% & P(Failure) = 0.87%(3)

P(Level4) = 1/3 × 1/9 × 1/27 × 1/81 = 0.000016935(4)

P(Success) = 0.0016935% & P(Failure) = 0.9983065%(4)

From Equation (3) we can see that for Level 3 there are 0.13% chances that an attacker will guess the password and when we proceed to Level 4 as shown in Equation (4), the probability value will decrease drastically, as for this level probability is 0.0016935% and it will keep on decreasing as the number of levels increases. With this less probability of success, there is extremely little chance that an attacker will guess the pattern. Therefore, this technique provides strong security against brute force, guessing and shoulder surfing attacks by using a very large password space.

## 5. Implementation and User Study

A prototype of the fractal-based graphical pattern, i.e., Sierpinski triangles, was implemented on the Android system. Although these devices have small screen sizes (which forces us to limit our levels to four in this study), this graphical pattern is not limited to Android devices, and it can be extended to any devices like Windows 8 ones, tablets, ATM machines, laptops, and personal computer locks, where authentication is required to operate the device. Since many handheld devices are powered by the Android operating system, therefore, we have developed and implemented our mechanism, i.e., FBAT on Android-based devices like smartphones. Usability tests have been conducted on different users’ Android phones after installing the application of the proposed technique to evaluate its usability and memorability. In this section, we discuss the implementation of FBAT on Android devices and the case study carried out on the same by describing the construction of the Sierpinski triangles, experimental design, participants, procedure and environment for the user study.

### 5.1. Implementation

Our Sierpinski triangle prototype was implemented with the Android SDK 4.0.3 Ice Cream Sandwich OS and API level 15. After developing a prototype, it was installed on users’ devices for further study.

### 5.2. Case Study

This section will discuss our user study, which includes experimental design, participants, procedure and environment for the study.

#### 5.2.1. Experimental Design

A real-time survey was conducted for evaluating the memorability and usability of FBAT:

(1) Memorability: This is the property which will determine how well a user will remember the created password. Will, they log in successfully after a period from registration or not?
(2) Usability: User experience was measured on the Sierpinski triangle, which includes successful login time, the time consumed during registration and login and the time used to reset in the registration and authentication phase.

During the experiment, 30 participants from various domains were invited and were asked to register their password in our scheme. Then they were asked to log in to the device after a week. Login after five attempts was considered as a failure. This number of the participant were selected as research [17] has shown that data from 30 users will be enough for an analysis. The usability of the proposed scheme was checked by calculating the registration and login time that each participant takes. It was performed to check whether our proposed scheme is time-consuming or not. The detailed procedure for collecting the results is described in Section 4 and the collected results are given in Section 6.

#### 5.2.2. Environment

The FBAT scheme was installed on users’ Android phones for the user study. However, the prototype was 1st tested on a Huawei P9 with a display size of 5.2 inches and 1080 × 1920 pixels resolution. This device had Android 7.0 (Nougat) version at the time of usability study of the prototype.

#### 5.2.3. Participant

Details of the participants are given in Table 3. Demographic information was collected using a questionnaire. The results show that there were 30 participants as prior studies did [17]. Participants were not familiar with the proposed technique, i.e., fractal-based graphical patterns or any other graphical pattern. The study group consisted of 5 university professors. 10 participants who were assistant professors (AP) and 15 lecturers. The majority of the lecturers and APs were selected from among non-computing-related departments because faculty members of the Computer Science and Information Technology (CS & IT) departments were considered technically strong and might have prior experience with graphical pattern techniques. As Table 3 shows, three professors were from the Biochemistry and Zoology department and two from CS & IT department with a specialization in human-computer interaction and information systems. Similarly, seven APs were from non-computing departments and three were from CS & IT departments. Ten lecturers were from science departments and five were from CS & IT.

#### 5.2.4. Procedure

This subsection will describe how the study was conducted in our experimental session:

(1) Introduction: In this phase, participants were explained the purpose of the study and a basic idea of fractal-based authentication. They were also shown an animation of using the proposed scheme so that they will get a better idea about it.
(2) Registration: In this phase, participants created a pattern while sitting in a private place to ensure safety during pattern creation. They were instructed in an introduction phase about password creation. Then told about selecting a triangle at each level using a color combination. Instructions were given to repeat the pattern to ensure the pattern, which will further save while pressing the confirm button. Instructions about retries were also given and during the registration phase, the user may click the retry button to change their chosen pattern.
(3) Login phase: After creating the pattern, participants were asked to log in or authenticate themselves on the system. They must follow the same pattern or sequence of triangles which they have selected during password creation.

Results are summarized in Table 3 above.

## 6. Results

The effectiveness of the proposed system was measured through a real-time survey and security analysis. The result representation theme is based on accuracy and usability. In this study, the perspective of accuracy is measured by the successful login rate and usability is measured with the average time spent on each fractal level. Result interpretation shows that proposed fractal-based pattern is practical to use in our daily life.

### 6.1. Accuracy

As mentioned earlier, accuracy is the successful login rate of the participants with the proposed scheme. As defined, successful login rate will be considered only if participants can successfully authenticate themselves within five attempts, otherwise it will be declared as a failure. We measure accuracy at two levels. Accuracy at first attempt is measuring $\sum_{fa}.$ as in Equation (5), whereas, the total accuracy, which includes both i.e., first attempt and attempts up to five levels is measured by $\sum_{t\_att}.$ as seen in Equation (6):

(5) $$\underset{fa}{\sum }=\frac{\sum_{sat}.}{\sum_{t\_att}.}$$

(6) $$\underset{t\_acc}{\sum }=\frac{\sum_{t\_Satt}.}{\sum_{t\_att}.}$$

where $\sum_{fa}=$ accuracy at first attempt, $\sum_{sat}=$ successful logins at first attempt, $\sum_{t\_att}=$ total number of attempts, $\sum_{t\_Satt}=$ total successful attempts and $\sum_{t\_acc}=$ total accuracy.

Table 4 below shows the results of the 30 participants who logged in successfully on the first attempt and their total login accuracy. The results depict that the percentage of total accuracy and accuracy of the first attempt is higher in the first session than the second session, as 29 out of 30 (96.66%) participants were able to log in successfully at the first attempt. Total accuracy was assumed to be 100% in the first session. It is because at that time they created a pattern and it was fresh in their mind, which helped them to log in at the first attempt. After a period of one week, participants were requested to use the application again with the same pattern which they created in the first session to unlock the device. Surprisingly, the results showed that 28 out of 30 (93.33%) participants were able to log in successfully. It includes 21 (70%) participants who logged in at the first attempt and six out of 30 (20%) who logged in after more than one attempt. Three participants (10%) took more than five attempts to log into the device. When we asked the participants about the extra attempts to login, they told that clicking on the triangles confused them because of the random motion of colors used in the pattern. They also stated that as information regarding bank account numbers etc. is confidential, and we used a complex pattern for this, it in turn increased the number of attempts required to crack the system. However, if we discuss the average successful login attempts of an individual person, it can be stated that the average single person took 3.8s for unlocking the device during the login phase. However, a few of the participants like those who were above 40 years of age took 6.1, 5.2,4.9, 7 and 5 s to unlock them. According to them, it was because three of the professors were above 55 years of age and they had weak eyesight issues and two of them selected a complex password.

Hence, from the survey results, it was concluded that the proposed FBAT is much easier to remember and use, besides its usability and memorability, it is difficult to breach for accessing the user’s confidential information. Users showed a positive response toward the usage, memorability and security strength of the FBAT and they highly appreciated the security parameters, i.e., random movement of color combinations and the level selection during password creation.

### 6.2. Usability

We calculated the total time consumed for successful login and registration to find the usability of our Sierpinski triangle pattern.

Total time consumed at registration and login phase is shown in Table 5. The average time consumed by the participants at registration was almost 1 min and 25 s. Average registration time is a bit lengthy. According to the survey conducted among the participants, it was found that 75% of participants said that the registration process is very easy. However, the rest of the participants said that they forgot to press the confirm button at the end of the registration, which results in an increased registration time. During the registration phase, participants have to select triangles from the pattern along with color combination, which they have to remember for the login session. After registration, participants were asked for authentication. Data collected from the participants shows that 45.5 s were required to log in to the device. As participants were not familiar with this technique, therefore, it results in extra time. However, participants were asked to use this pattern as a lock for a week. After the specific time of one week, participants were requested to participate in an experiment. Data collected after a week was surprising. As shown in Table 3. It revealed that, after continuous usage of one week, time for login reduced to 20.3 s. In the third phase, that was conducted after 2 weeks, again, data were collected from the participants. The result was surprising in that time consumed for the login session after two weeks was drastically reduced to 4.4 s. It will keep on reducing after spending a little bit extra time on the pattern and 79% of the participants said that spending a little bit extra time on the registration phase was worthwhile if the authentication scheme will help in the protection of passwords from the shoulder surfing technique.

## 7. Security Analysis

This section will discuss the security analysis of our proposed authentication system i.e., FBAT against attacks like shoulder surfing and brute force attacks by comparing with existing techniques used for overcoming these attacks.

Comparisons of FBAT with existing techniques like Mod 10 [26], STL method [27], FC method [28], BW method [29]and MobSecure [30] were used to perform the analysis in the context of security, login average time, unlock percentage in brute force context and password space. Password space states the total combination of keys used for creating the password. The results represent the password space of FBAT up to Level 4 like other techniques. Security and usability in the context of authentication, ease of use and remembering of the pattern were considered as a priority in comparative analysis. Hence, according to Table 6, it can be deduced that FBAT is robust and strong in all selected scales used for comparisons, i.e., average login time, unlocking percentage and password space.

### 7.1. Breaching FBAT

To check the breaching strength of FBAT, if it exists, multiple experiments were conducted by dividing our participants into three different teams, Alpha, Beta, and Gamma. Each team has ten randomly selected members. Random member from team Alpha were selected to create a password by selecting one level of FBAT. Similarly, members of the Beta team created a password by selecting two levels of FBAT and members of the Gamma team created a password by selecting three levels of FBAT. Password creation activity was performed in full isolation. Each member created a password by selecting color combinations within FBAT. Every time, the color changed randomly whenever it the authentication step is entered. In order to check the strength of patterns in every aspect, after ten attempts by each team member, e.g., Alpha, Beta, and Gamma, the pattern was then swapped among all teams such that, all team members will get a chance to crack the passwords of each level i.e., Level 1, Level 2 and Level 3. Participants were asked to identify/crack the pattern.

Feedback from the participants was collected, and it showed that no one was able to crack the passwords at levels2 and 3. However, at Level 1, three members from team Alpha, three members from team Beta and three members from team Gamma were successful in cracking the password. Hence, we infer from the analysis that Level 2 can be used in situations where minimal security is required and for scenarios that require high-security, users are recommended to use a maximum level password. It can be seen from the analysis that each participant had a maximum number of attempts to crack the password, but they were still successful in cracking the password at Level 1. This is because, according to the Equation (2) there were 3.7% chances that an attacker will crack the password. However, our proposed technique still has better security, because when the attacker tries to breach the pattern, after 5th attempt, FBAT will disable the pattern for 30 s due to which all the patterns which the attacker remembers will by fully shuffled creating more difficulty to the attacker. 

In the experimental phase, there were nine participants in each group and each participant had thirty chances to crack the password at each level (i.e., ten at Level 1, ten at Level 2, ten at Level 3). Hence, every team had 270 guessing attacks to crack the password at all levels as shown in Table 7. Three members of team Alpha cracked the password at the 7th, 6th and 7th attempt, three members of team Beta guessed the password at the 5th, 8th and 6th attempt and three members of team Gamma identified the level 1password after the 7th, 5th and 9th attempt. Those participants who were successful in cracking the password at level 1, did not need the remaining attempts. Therefore, subtracting those attempts results in a total of 250 attempts at Level 1.

Our proposed technique was further tested against the possible threats like shoulder surfing, smudge attack, and brute force attack. These attacks are further explained below.

### 7.2. Shoulder Surfing

Shoulder surfing is a real threat to any kind of authentication system like graphical or textual-based password authentication schemes. To protect against this attack, a lot of new authentication techniques were proposed for protecting the system from different attacks. However, most of the proposed schemes do not overcome this attack, if shoulder surfing is done using camera recording. For example, proposed techniques like spying resistant keyboards [31,32] and PIN-entry methods [29] are based on the issues of short-term memory difficulties. The camera-based technique can easily be used to crack the passwords of these schemes in a shoulder surfing attack.

The proposed Sierpinski triangle-based technique (i.e., FBAT) used for authentication process helps in overcoming shoulder surfing attacks. In the proposed scheme, the user created a password by selecting different triangles with the help of color combinations. In every stage of the authentication, besides the one color triangle that was selected as a password, all the other triangles will be decoy triangles in a big triangle. Color combinations randomly shuffle in every stage of the password authentication depending on which user opted for a triangle of relevant color which was used during the password creation phase. Random movement of colored triangles in every phase of authentication forces the user to select their relevant triangle in a big triangle. Furthermore, the login indicator for each Sierpinski triangle varies because of the randomness of the color scheme. Therefore, touching an area on the screen also changes at every stage. However, besides the random movement of color triangles, the increase in levels at every stage of FBAT reduces the area of each triangle and increases the perimeter. Therefore, due to the random movement of colors and reduction in area of triangles the size of the patterns becomes small, due to which, while selecting the pattern, the maximum triangles beneath the thumb or finger are hidden, which makes FBAT secure against shoulder surfing. Table 6 represents that the password space at Level 4is much higher in FBAT as compared to other techniques used for the comparisons and the password space will keep on increasing with the increase in levels.

### 7.3. Smudge Attack

Residues left on the screen are known as a smudge. Therefore, attacks based on this feature are known as smudge attacks [13]. The attacker tries to capture sensitive information by observing the smudges left on the screen by the recent users. In the proposed FBAT scheme, the triangles on each level increase with the random combination of colors. Smudges can reveal the passwords effectively only in pattern-based approaches, If the password is PIN-based then the accuracy of smudges will be reduced. Therefore, due to the random nature of colors in FBAT, the user will have to hit the triangles on different locations on the screen. This will produce a maximum smudge on the screen at a different location because of the randomness of the colors in the pattern. Hence, the amount of smudge left by the user on the screen will not provide any useful information to the attacker because of the randomness of the login indicator.

### 7.4. Brute force Attack

Checking all possible combinations for cracking a password is known as a brute force attack [1] [33,34]. It is performed until and unless attackers become successful in cracking the password. Our proposed technique has a very low possibility for cracking the pattern. As Equation (3) explains there is only a 0.13% chance of cracking the password. However, if we increase the level of the Sierpinski triangle, password complexity will increase by the Equation (4), i.e., 0.0016935%, and the complexity will keep on increasing by increasing the level of the technique. Passwords are created by the combination of different colors. Hence, selecting different color combinations during brute force attempts will be another challenge because of the random motion of colors. Our two-pattern detection, i.e., selecting accurate triangles with accurate colors will reduce the cracking attempts at each level, as the attacker has to make combine accurate color and accurate triangle selection at every stage. Brute force attack is considered the most challenging technique. However, only a limited number of attempts will be available for cracking the password. After five attempts, the account will be blocked. To unlock the account, the user will have to enter the secret PIN number. Therefore, our proposed technique will overcome all the challenges that occurred in security.

## 8. Conclusions

With the increasing trend of smart devices, i.e., wireless technology and mobile apps, etc. people started moving toward them. Ubiquitous computing helps access different applications anytime and anywhere. Therefore, users have started using those applications to save their confidential information in them, e.g., account numbers, passwords and personal pictures. Hence these digital devices need some sort of authentication system, to protect their valuable information. However, the authentication process in public is still vulnerable to shoulder surfing attacks. Even the complicated passwords can be cracked through shoulder surfing. Usually, users secure their devices using a textual password or PIN number. However, these traditional techniques are vulnerable.

To overcome shoulder surfing, brute force and smudge attacks, this study proposed a novel technique based on fractal i.e., Sierpinski triangle. Due to the property of Sierpinski triangles, their size will keep on reducing by increasing the levels and randomness of colors will give no clue to the attacker for cracking it. Complexity level of FBAT scheme keeps on increasing by increasing the levels of fractal. Experimental results indicate that total accuracy of login attempts after a period of one week is 93.33% as described in the accuracy section earlier. However, from the perspective of usability, the total time consumed in login attempts at the registration phase and its mean is 85.4 s whereas, the login time was reduced drastically with a mean of4.4 s by the third attempt. 

Hence, according to the results, it is considered that the proposed technique is usable and easy to use for the authentication of a system. It will reduce shoulder surfing attacks. The proposed technique can be implemented for any device requiring authentication like ATM machines, mobile devices, and laptops, etc. The results show that the proposed technique is practical in a real-life scenario.

## Figures and Tables

**Table 1:** Password space of proposed technique up to level 5.

Level	¢	B	ȿ = ϐ^¢^
L1	1	3	3
L2	2	9	81
L3	3	27	19,683
L4	4	81	43,046,721
L5	5	243	847,288,609,443

**Table 2:** Notations.

Ю	Object of string pattern	
ς	Re attempt variable	
ϸ	Pattern Variable	
∂	Permanent storage	
ϐ	Number of possible symbols for password creation	
¢	Length of password	
ȿ	Password space	
L1	{1,2,3}	numbers of triangles at level 1
L2	{1,2,3,4, ……….., 9}	numbers of triangles at level 2
L3	{1,2,3,4, ……..., 27}	numbers of triangles at level 3
L4	{1,2,3,4, ………, 81}	numbers of triangles at level 4
L5	{1,2,3,4, ……, 243}	numbers of triangles at level 5

**Table 3:** Demographic Information.

Demographic Information	Category	Frequency	Percentage
Gender	Male	17	56.66%
	Female	13	43.33%
Age	25–30	18	60.00%
	30–35	5	16.66%
	35–40	2	6.66%
	Above 40	5	16.66%
Designation	Professor	5	16.66%
	Assistant Professor	10	33.33%
	Lecturer	15	50.00%
Department	Computing	10	33.33%
	Non-computing	20	66.66%

**Table 4:** Authentication accuracy in two sessions.

	First Attempt	Between 1–5 Attempts	>5 Attempts
Login (1st time)	100%		
Login (after week)	70%	20%	10%

**Table 5:** Average time in seconds at login and registration phase.

	Registration (Reg.)/Login		
	Mean	Median	S. D
Total Time (s) Reg.	85.4	60.3	40.57
Total Time (s) Login (1st)	45.4	55.5	31.6
Total Time (s) Login (2nd)	20.3	16.2	9.5
Total Time (s) Login (3rd)	4.4	2.33	1.45

**Table 6:** Comparative analysis of Mod 10, STL, BW, FC and MobSecure with FBAT.

Techniques	Resilient Against	Average Login Time	Percentage of Error to Unlock	Password Space
Mod 10	Strong Adversary	9.2	16.66	10^4^
STL	Strong Adversary	13.41	9.09	10^4^
BW	Weak adversary	19.5	9.09	10^4^
FC	Weak adversary	20.31	13.04	10^4^
MobSecure	Weak adversary	20.31	13.04	10^4^
FBAT	Strong adversary	4.4	1.6935 × 10^−5^	81^4^

**Table 7:** Breach Complexity of FBAT.

Levels	Total Attempts	Unsuccessful Attempts	Successful Attempts
Level 1	270	241	9
Level 2	270	270	0
Level 3	270	270	0
